# A near-infrared spectroscopy routine for unambiguous identification of cryptic ant species

**DOI:** 10.7717/peerj.991

**Published:** 2015-09-15

**Authors:** Martin-Carl Kinzner, Herbert C. Wagner, Andrea Peskoller, Karl Moder, Floyd E. Dowell, Wolfgang Arthofer, Birgit C. Schlick-Steiner, Florian M. Steiner

**Affiliations:** 1 Molecular Ecology Group, Institute of Ecology, University of Innsbruck, Innsbruck, Austria; 2 Institute of Applied Statistics and Computing, University of Natural Resources and Life Sciences, Vienna, Austria; 3 Agricultural Research Service, United States Department of Agriculture, Manhattan, KS, USA

**Keywords:** Cryptic-species complex, Ants, Formicidae, Neural networks, One-vs-all strategy, Partial least squares regression, Random forests, Species identification tool, *Tetramorium*

## Abstract

Species identification—of importance for most biological disciplines—is not always straightforward as cryptic species hamper traditional identification. Fibre-optic near-infrared spectroscopy (NIRS) is a rapid and inexpensive method of use in various applications, including the identification of species. Despite its efficiency, NIRS has never been tested on a group of more than two cryptic species, and a working routine is still missing. Hence, we tested if the four morphologically highly similar, but genetically distinct ant species *Tetramorium alpestre*, *T. caespitum*, *T. impurum*, and *T.* sp. B, all four co-occurring above 1,300 m above sea level in the Alps, can be identified unambiguously using NIRS. Furthermore, we evaluated which of our implementations of the three analysis approaches, partial least squares regression (PLS), artificial neural networks (ANN), and random forests (RF), is most efficient in species identification with our data set. We opted for a 100% classification certainty, i.e., a residual risk of misidentification of zero within the available data, at the cost of excluding specimens from identification. Additionally, we examined which strategy among our implementations, one-vs-all, i.e., one species compared with the pooled set of the remaining species, or binary-decision strategies, worked best with our data to reduce a multi-class system to a two-class system, as is necessary for PLS. Our NIRS identification routine, based on a 100% identification certainty, was successful with up to 66.7% of unambiguously identified specimens of a species. In detail, PLS scored best over all species (36.7% of specimens), while RF was much less effective (10.0%) and ANN failed completely (0.0%) with our data and our implementations of the analyses. Moreover, we showed that the one-vs-all strategy is the only acceptable option to reduce multi-class systems because of a minimum expenditure of time. We emphasise our classification routine using fibre-optic NIRS in combination with PLS and the one-vs-all strategy as a highly efficient pre-screening identification method for cryptic ant species and possibly beyond.

## Introduction

Correct species identification is crucial for most fields of biology, including biodiversity research, conservation biology, invasion biology, and the understanding of evolution (Bickford et al., 2007; Pfenninger & Schwenk, 2007). Species with very subtle morphological differences relative to other species, termed cryptic species (Bickford et al., 2007), pose a challenge for classical taxonomy and species identification. Cryptic species are known from all biogeographical regions and from all major metazoan taxa (Pfenninger & Schwenk, 2007). Estimation of crypsis across the animal kingdom is difficult, but in some groups more than 50% of species are morphologically hardly discriminable (Seifert, 2009). Moreover, complexes of cryptic species, i.e., more than two species not differentiable, are not a rarity in insects (Hebert et al., 2004; Smith et al., 2008; Seifert, 2009), in other arthropods (Wilcox et al., 1997; Arthofer et al., 2013), and even in vertebrates (Oliver et al., 2009). One major problem for the in-depth investigation of cryptic species is the high effort needed for correct species identification.

Misidentifications are not a rarity in ecological studies and can lead to error cascades in biology. Far-reaching consequences are, for example, wrong interpretations of biological studies, inaccurate environmental management, and loss in biodiversity (Bortolus, 2008). Moreover, errors in species identification can produce high costs in economy, such as, when imported goods are discarded because of the presence of organisms erroneously identified as pest species (Boykin et al., 2011). Thus, a 100% identification certainty, i.e., a residual risk of misidentification of zero within the available data, is highly desirable for a species identification routine to allow correct downstream investigations and to avoid unwanted consequences (Bortolus, 2008).

Near-infrared spectroscopy (NIRS) is, among other applications, a technique for species identification (Rodriguez-Fernandez et al., 2011). The efficiency of NIRS as a fast and inexpensive method for the classification of substances differing in chemical composition has been shown in numerous studies (e.g., Foley et al., 1998; Rodriguez-Fernandez et al., 2011), and it has been applied to medicine (Quaresima, Lepanto & Ferrari, 2003), pharmacology (Reich, 2005), soil science (Chang et al., 2001), landscape ecology (Youngentob et al., 2012), biotechnology (Balabin & Safieva, 2011), vector control (Sikulu et al., 2010), and the agriculture and food industry (Williams & Norris, 2001). NIRS analysis of chemical compounds from insect surfaces, which comprise mainly a variety of cuticular hydrocarbons (CHCs), has been multiply used to discriminate sex, age, infection status with certain bacteria, and population of origin (Newey, Robson & Crozier, 2008; Aw, Dowell & Ballard, 2012) as well as to identify species (Cole et al., 2003; Fischnaller et al., 2012).

NIRS generates large sets of raw data, and a critical selection of the most appropriate analysis approach is essential to extract data subsets informative for a specific purpose, e.g., for classification problems (Pasquini, 2003). One prominent method for the analysis of NIR spectra is partial least squares regression (PLS), a combination of principal component analysis and multiple linear regression (Abdi, 2010). It has been used for the analysis of NIR spectra for species identification (e.g., Jia et al., 2007; Fischnaller et al., 2012) because of its capability to handle data with many more variables than observations (Pasquini, 2003).

The use of machine learning algorithms is an alternative in resolving species identification problems (Clark, 2003; Gaston & O’Neill, 2004). Characteristics of artificial neural networks (ANN) are their capability to learn from observations and to perform non-linear multivariate data mining for pattern recognition (Clark, 2003). Dowell et al. (1999) and Aldrich et al. (2007) showed that ANN are suitable for species discrimination using NIRS data. However, ANN cannot efficiently handle data sets with many variables and few observations, and thus a prior reduction of variables is necessary (Svetnik et al., 2003; Liu et al., 2013).

Another category of machine learning algorithms is random forests (RF). This method uses ensembles of decision trees for classification, regression, or unsupervised analysis (Breiman, 2001). Some of the major advantages of RF are the handling of data sets with large variable and small observation numbers and the avoidance of model overfitting (Breiman, 2001). RF has been shown to be very efficient for classification problems, giving more accurate results than other methods (Svetnik et al., 2003; Liu et al., 2013) and for tackling biological questions, including via spectral data (Menze et al., 2009; Lee et al., 2012).

**Figure 1:**
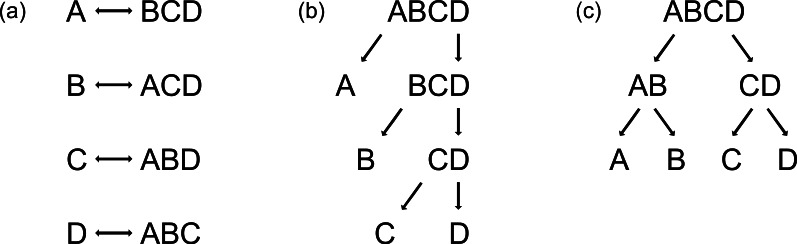
Three possibilities to reduce multi-class systems into two-class systems. (a) One-vs-all, (b) binary-decision type A, and (c) binary-decision type B strategy.

ANN and RF are able to handle data consisting of more than two classes, but PLS is not, and thus the reduction of multi-class problems into two-class problems is necessary. This can simply be achieved by using either the one-vs-all strategy (Rifkin & Klautau, 2004) (Fig. 1a) or binary decision trees (Figs. 1b and 1c). By applying the one-vs-all strategy, one group (Class 1) is compared with the pooled set of the remaining groups (Class 2), each group alternately being Class 1 (Fig. 1a). Binary decision trees can be applied either as sequential classification of one group versus all others with a decreased number of groups after every step (Fig. 1b, here named binary-decision type A), or by first comparing two subclasses and then classifying the groups of each subclass in pair-wise comparisons (Fig. 1c, here named binary-decision type B). The optimisation of the chronology of class separation, i.e., the order in which classes are split off to gain maximum identification success, requires exhaustive testing of all possible combinations.

To test NIRS reliability, we have chosen the four species of the cryptic *Tetramorium caespitum*/*impurum* species complex (Hymenoptera: Formicidae) that co-occur above 1,300 m above sea level (a.s.l.) in the Alps. In total, the cryptic-species complex consists of at least seven species in Central Europe (Schlick-Steiner et al., 2006); the three species not included here are restricted to lower altitudes. All species are morphologically highly similar, but are known to vary in their CHC profiles, a fact which has already been used for species delimitation and discrimination (Schlick-Steiner et al., 2006; Klarica et al., 2011).

For an unambiguous identification of groups containing more than two cryptic species, the efficiency of NIRS as an alternative to conventional identification methods and the performance of PLS, ANN, and RF have not been investigated yet. Hence, we tested whether a complex of more than two cryptic species with similar ecological requirements can be identified with 100% certainty using fibre-optic NIRS. Additionally, we examined which of the three strategies, one-vs-all or binary-decision type A, or B, is best to address multi-class problems when not more than two classes can be analysed in parallel (e.g., using PLS).

## Materials and Methods

### Study system

Four of the seven cryptic ant species from the *Tetramorium caespitum*/*impurum* complex (*T. alpestre* Steiner, Schlick-Steiner & Seifert, 2010, *T. caespitum* (Linnaeus, 1758), *T. impurum* (Foerster, 1850), and *T*. sp. B *sensu* Schlick-Steiner et al. (2006)) were selected for this study because of their occurrence in the same habitat above 1,300 m a.s.l. in the Alps (Steiner et al., 2010) and their similar ecological requirements. Specimens were collected from nests in a large geographic area, from Spain to Armenia and from Finland to Greece (Table 1 and Table S1) between 1993 and 2012. Individuals were submerged in absolute ethanol at the collection site and afterwards stored at 4 °C or −20 °C. The number of specimens stored at each of the two temperatures was balanced among the species. Nests in spatial vicinity of less than 1 km were treated as belonging to the same population.

**Table 1:** Sample information of the four species with number of populations, longitudinal, latitudinal, and altitudinal extensions.

	Pop	Lon		Lat		Alt	
		min	max	min	max	min	max
***T. alpestre***	29	6.40	13.95	41.69	47.22	1,300	2,400
***T. caespitum***	45	−2.38	27.27	41.81	59.83	2	1,400
***T. impurum***	45	−3.28	26.35	40.10	50.99	7	2,000
***T.* sp. B**	45	9.80	44.02	37.95	50.92	100	1,950

**Notes.**

Pop Number of populations. Nests in spatial vicinity of less than one kilometre from each other were treatd as one population Lon Minimum and maximum longitudinal position in decimal format, positive values indicate position east of Greenwich, negative values indicate position west of Greenwich Lat Minimum and maximum latitudinal position in decimal format Alt Minimum and maximum altitudinal position in m above sea level

Workers were dry-mounted by first removing the ant’s gaster (abdomen behind the waist) for subsequent genetic analysis and then gluing the tibiae and tarsi on a white paper card using customary wallpaper adhesive, so that a specimen’s dorsal and lateral surface was accessible for NIRS measurements. Dry-mounted specimens were stored at room temperature in glass-covered insect boxes avoiding intensive exposure to light, dust, and moisture for at least one month before NIRS measurements.

DNA extraction from the gaster of one individual per nest and PCR amplification of a mitochondrial cytochrome c oxidase subunit I (COI) gene stretch for species identification followed the protocol of Steiner et al. (2005) with one slight modification, i.e., addition of 2.25 µl bovine serum albumin (0.2 µg/µl) to the PCR reaction mix. Additionally, out of the 176 nests analysed by NIRS, each two individuals from 108 nests and one individual from 22 nests were identified using traditional morphometrics applying the method of Steiner, Schlick-Steiner & Moder (2006) and Steiner et al. (2010). Morphometric analysis of individuals from all nests would have required prohibitive amounts of time (see Discussion). In none of those instances where the species ID had been determined by more than one independent method did we obtain conflicting results, as would be expected from, for example, hybridisation. Detailed information on the specimens used in this study is given in Table S1. New sequences have been submitted to GenBank under the accession numbers KT248392–KT248508.

### NIR data collection

Spectral data were collected using a Labspec^®^ 5000 Portable VIS/NIR Spectrometer (ASD inc., Boulder, Colorado, USA) with a wavelength range from 350 to 2,500 nm and 1 nm resolution. Before each measurement session, a reference baseline was created by positioning the 3 mm diameter bifurcated fibre-optic probe uprightly 2.2 mm above a white Spectralon^®^ plate; this distance for optimum baseline acquisition was determined empirically by measuring the reflectance at increasing plate-probe distances starting from 0.1 mm until the reflectance reached maximum intensity.

For measurements, mounted ants were placed above a Spectralon^®^ plate using a goniometer-style pin-holding stage. The probe was positioned uprightly 2.2 mm above the dorsal surface with focus on the head and mesosoma. For *T. caespitum*, *T. impurum*, and *T*. sp. B, 135 workers from 45 nests (three specimens per nest), and for *T. alpestre*, 123 workers from 41 nests were measured, resulting in spectra from a total of 528 insects. All measurements were performed in the same laboratory under constant artificial illumination (4,000 K, 2.9 µmol m^−2^ s^−1^). Relative humidity and air temperature were kept at 36% and 22.5 °C, respectively. To increase the signal-to-noise ratio, the average spectrum of 50 measurement replicas was collected for each specimen.

### NIR data analysis

Spectra were converted to the Galactic Spectrum file format (.spc) and automatically mean-centred using ASDtoSPC version 5.6 (ASD Inc.). Regions below 500 nm and above 2,300 nm were removed because of high noise levels caused by sensor and lighting limitations (Dowell, Noutcha & Michel, 2011).

For each of *T. caespitum*, *T. impurum*, and *T*. sp. B, 30 nests were selected randomly for the computation of the calibration model; for *T. alpestre*, 26 nests were used for calibration. The remaining 15 nests were used as an independent validation set for testing the models. All nests of a population were assigned either to the calibration or to the validation set to assure the independency of the validation set.

The inability of PLS to handle more than two groups necessitated the reduction of the multi-class system to a two-class system. The three possibilities of doing so, one-vs-all strategy, binary-decision type A, and binary-decision type B, were compared by estimating the times needed for elaborating the PLS calibration models. In doing so, we calculated the overall times needed for an exhaustive search under each strategy; an exhaustive search is the only approach by which the optimal calibration model for every decision step in the species identification process can be identified. For every possible combination of species, we used the empirical value of 1.33 h for calibration-model elaboration, which in our experience is realistic after initial training. This time estimation includes the evaluation of, on average, 13 different PLS factors.

For the one-vs-all strategy, given *c* classes, the number of model-elaboration steps *s* is calculated as

(1) \begin{eqnarray*} {s}_{c}=\left\{\begin{array}{c} \displaystyle c-1~\forall ~c=2\\ \displaystyle c~\forall ~c\geq 3. \end{array}\right. \end{eqnarray*}

For the binary-decision type A, the number of computation steps follows

(2) \begin{eqnarray*} {s}_{c}=({2}^{(c-1)}c)-c\frac{(c+1)}{2}. \end{eqnarray*}

The calculation for binary-decision type B is more complex, as the calculation of the number of combinations requires different equations for odd and even numbers of classes. First, the number of levels *n_l_* to repeatedly halve *c* classes is calculated by *n_l_* = int(log_2_(*c*)). At each level, a number of groups *n_i_*, containing all classes, exists. Now let *x*_*i*,*k*_ be the number of classes at level *i* in group *k* and define the auxiliary variables *v*_1,*k*_ = int((*x*_*i*,*k*_ + 1)/2) and *v*_2,*k*_ = *x*_*i*,*k*_ − *v*_1,*k*_. The total number of computation steps then calculates by the sum of the binomial coefficients

(3) \begin{eqnarray*} \displaystyle {s}_{c}&=&\displaystyle \left(\begin{array}{c} \displaystyle c\\ \displaystyle \mathrm{int}(c/2) \end{array}\right)+\left(\begin{array}{c} \displaystyle c\\ \displaystyle 2 \end{array}\right)\nonumber\\ \displaystyle &&\displaystyle +\, \left\{\begin{array}{ll} \displaystyle \sum _{i=1}^{{n}_{l}}\sum _{j=1}^{{n}_{i-1}}\left(\begin{array}{c} \displaystyle c\\ \displaystyle {v}_{1,k} \end{array}\right)\left(\begin{array}{c} \displaystyle c-{v}_{1,k}\\ \displaystyle {v}_{2,k} \end{array}\right)~/~2&\displaystyle \forall {v}_{1,k}={v}_{2,k} \\ \displaystyle \sum _{i=1}^{{n}_{l}}\sum _{j=1}^{{n}_{i-1}}\left(\begin{array}{c} \displaystyle c\\ \displaystyle {v}_{1,k} \end{array}\right)\left(\begin{array}{c} \displaystyle c-{v}_{1,k}\\ \displaystyle {v}_{2,k} \end{array}\right)~&\displaystyle \forall {v}_{1,k}\not = {v}_{2,k} \end{array}\right.\nonumber\\ \displaystyle &&\displaystyle +\, \left\{\begin{array}{ll} \displaystyle \sum _{i=1}^{{n}_{l}}\sum _{j=1}^{{n}_{i-1}}\left(\begin{array}{c} \displaystyle c\\ \displaystyle {v}_{1,k+1} \end{array}\right)\left(\begin{array}{c} \displaystyle c-{v}_{1,k+1}\\ \displaystyle {v}_{2,k+1} \end{array}\right)~/~2&\displaystyle \forall {v}_{1,k+1}={v}_{2,k+1} \\ \displaystyle \sum _{i=1}^{{n}_{l}}\sum _{j=1}^{{n}_{i-1}}\left(\begin{array}{c} \displaystyle c\\ \displaystyle {v}_{1,k+1} \end{array}\right)\left(\begin{array}{c} \displaystyle c-{v}_{1,k+1}\\ \displaystyle {v}_{2,k}+1 \end{array}\right)&\displaystyle \forall {v}_{1,k+1}\not = {v}_{2,k+1}. \end{array}\right. \end{eqnarray*}

**Figure 2:**
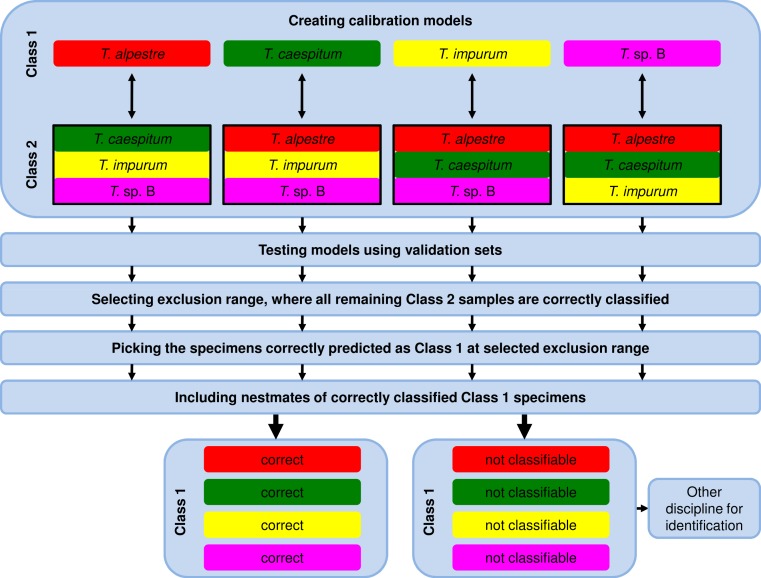
Workflow of data analysis using the one-vs-all strategy. Models were elaborated, and optimum models were selected and tested using the validation-set prediction accuracy. An exclusion range of prediction values was selected, where all remaining specimens of the Class 2 validation set were correctly classified (exclusion of false positives). As a consequence, the remaining correctly classified Class 1 validation-set specimens (true positives) were unambiguously identified. Nestmates of the recently identified specimens were also treated as correctly identified. All excluded and thus not identified Class 1 validation-set specimens were unidentifiable using NIRS and the one-vs-all approach. Another method for identification is necessary.

Due to the fact that the model-elaboration times for the binary decision strategies were higher than for the one-vs-all strategy (see Results), we used exclusively the one-vs-all strategy for further analyses by comparing the calibration set of one species (Class 1, Fig. 2) with the pooled calibration set spectra of the remaining three species (Class 2). In testing the model, each specimen of the independent validation set received a prediction value from PLS, ANN, and RF. Individuals with a value ≤1.5 with PLS or ≤0.5 with ANN and RF were assigned to Class 1 and ones with a value >1.5 with PLS or >0.5 with ANN and RF to Class 2.

**Figure 3:**
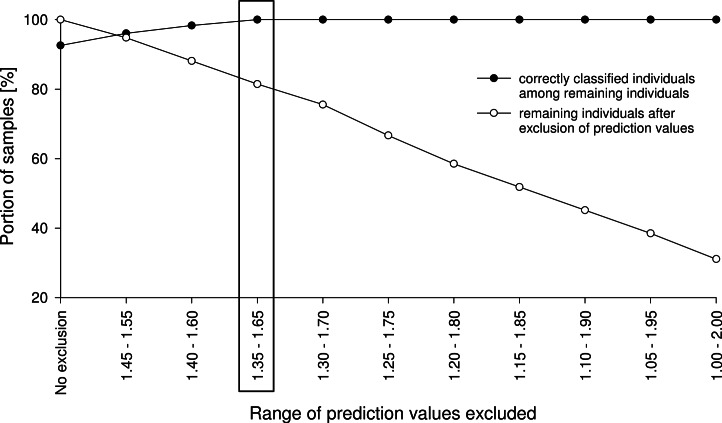
Example of an exclusion plot of Class 2 validation-set individuals. With increasing range of exclusion, more individuals were excluded from prediction and more remaining individuals were correctly predicted. In this example, by excluding all specimens with prediction values from 1.35 to 1.65, all remaining specimens were correctly classified.

For the 100% correct classification of Class 1 validation-set specimens, we sought models which resulted in the correct classification of all Class 2 validation-set specimens, i.e., in the elimination of all Class 2 validation specimens incorrectly predicted as Class 1 (false positives). Hence, individuals with a prediction value in an interval around 1.5 with PLS or 0.5 with ANN and RF were excluded by augmenting this interval in 0.1 incremental steps (1.45–1.55, 1.4–1.6, etc.) until no false-positive specimens from validation set Class 2 remained (Fig. 3). At that point, the Class 1 prediction pool was without Class 2 individuals and as a consequence, all retained individuals predicted as Class 1 were correctly classified. Finally, based on the assumption that nests contain only specimens of one species, individuals excluded during this procedure were treated as identified if at least one nestmate was among the correctly classified Class 1 specimens. This procedure was repeated for all species, alternately treating each of them as Class 1. All individuals not identified during this approach were considered as unclassifiable using NIRS; an alternative discipline would be necessary for species discrimination.

Principal component analysis (PCA) was calculated with PAST version 2.17 (Hammer, Harper & Ryan, 2001) using the reflectance values for 1,801 wavelengths as variables. PLS regression was performed using the software Grams AI and PLSplus/IQ version 8.0 (Thermo Electron Corporation, Salem, New Hampshire, USA) for the classification of species. The number of factors used for the computation of the model and the quality of the calibration model were determined by visual evaluation of the regression coefficient and the correct classification rate of the validation set (i.e., the prediction values).

ANN were built as backpropagation feed-forward networks using the software NeuroShell Classifier version 3.0 (Ward Systems Group Inc., Frederick, Maryland, USA). Due to the fact that ANN were not able to use all 1,801 variables, 150 were randomly selected, starting from 500 nm with 12 nm increments, similar to the procedure of Aldrich et al. (2007). The NeuroShell Classifier calculated the optimum number of hidden neurons for each model.

The RF analysis was performed using the software package randomForest version 4.6–7 in R (Liaw & Wiener, 2002) with 1,000 bootstrapping specimens (ntree) and default settings for the number of variables used for searching the best split at each node (mtry) for 1,801 and 150 variables, in analogy to the PLS and ANN analyses, respectively. The selection of the optimum parameters was done by exhaustively evaluating parameter combinations. Although ANN and RF are able to handle more than two classes in parallel, their performance in doing so was insufficient for our aim of unambiguous identification (Table S2). Thus, we only refer to the one-vs-all strategy in the following.

All computations were performed on a personal computer equipped with an AMD A6-3400M processor with 1.40 GHz and 8 GB RAM using 64-bit Microsoft^®^ Windows^®^ 7 as operating system. Computation time estimations refer to this system configuration.

## Results

**Figure 4:**
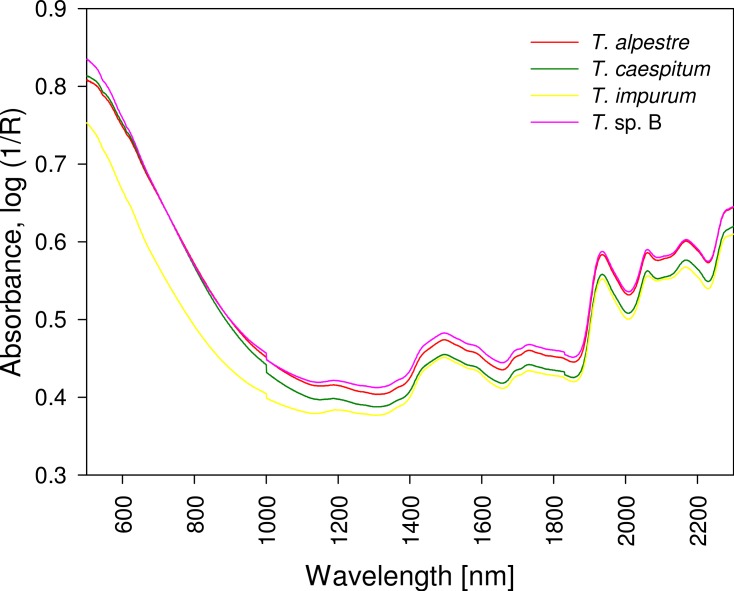
Mean spectra of four cryptic *Tetramorium* species. All spectra showed a similar curve progression, and differences were not detectable by visual evaluation; chemometric analysis was required. R, reflectance.

**Figure 5:**
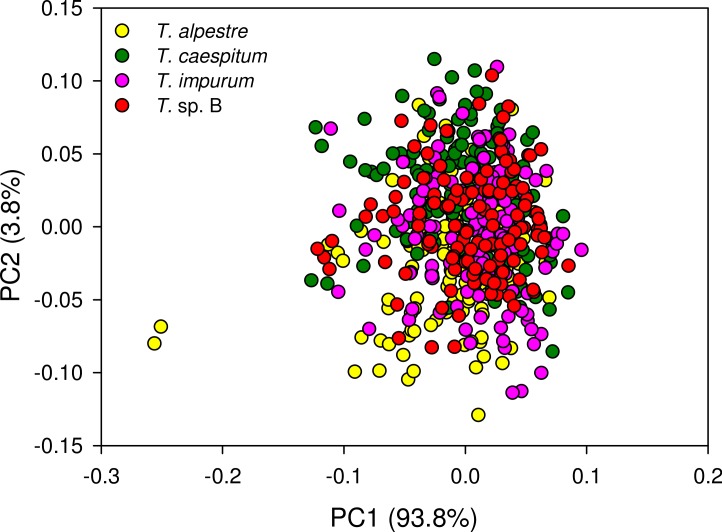
Principal component analysis scatter plot of 1,801 spectral variables from all 528 specimens. Percentage of the variation explained by each of the first two principal components (PC) given. There was no indication of clustering.

The four cryptic species largely overlapped in the individual spectra. In the mean spectra, some offset was discernible across species (Fig. 4), but no species-specific regions were found by visual inspection. The PCA plot showed no distinct clustering of the spectral data according to species (Fig. 5).

**Figure 6:**
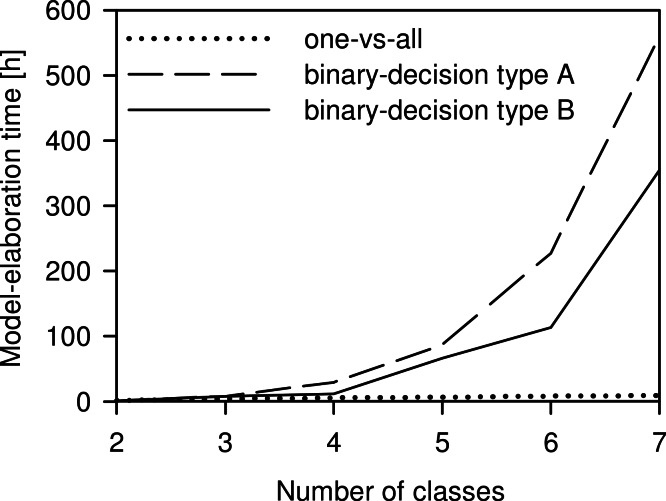
Comparison of the time needed for model elaboration. Time for model elaboration for all possible combinations with increasing numbers of classes using the one-vs-all, the binary-decision type A, and the binary-decision type B strategy based on 1.33 h of working time per model. The maximum number of classes analysed was seven, as represented by the *Tetramorium caespitum*/*impurum* complex in Central Europe (Schlick-Steiner et al., 2006).

The model-elaboration times differed across the three strategies of reducing a multi-class system to a two-class system (Fig. 6). For the four-class system, as used in this study, the estimated elaboration times were 5.3 h for one-vs-all, 29.3 h for binary-decision type A, and 12 h for binary-decision type B. These differences increased with increasing number of classes, e.g., for a seven-class system as represented by all Central European species of the *Tetramorium caespitum*/*impurum* complex, one-vs-all would take 9.3 h, binary-decision type A 560.0 h (ca. 14 weeks), and binary-decision type B 354.7 h (ca. 9 weeks). Hence, exclusively the one-vs-all strategy was used for further analyses in this study.

Prior to the exclusion of prediction values, specimens were classified with an average error rate across all species of 58.3% using PLS (min. 42.2%, max. 80.0%, Table 2), 42.8% using ANN (min. 22.2%, max. 57.8%), and 92.2% (min. 91.1%, max. 93.3%) and 78.9% (min. 68.9%, max. 95.6%) using RF with 150 and 1,801 variables, respectively. After the exclusion of specimens within the excluded prediction-value range, i.e., eliminating all false positives and thus reaching 100% certainty, PLS unambiguously identified 21 *T. alpestre* workers (46.7% of 45 workers), three *T. caespitum* workers (6.7%), eight *T. impurum* workers (17.8%), and two *T*. sp. B workers (4.4%). After including the nestmates of the correctly identified specimens in the pool of correct classifications, the portion of unambiguously identified specimens increased to 66.7% (*T. alpestre*), 20.0% (*T. caespitum*), 46.7% (*T. impurum*), and 13.3% (*T*. sp. B). Across all species, 22 nests (66 individuals, 36.7%) were correctly identified without uncertainty.

**Table 2:** Species classification results for the classification of the validation set using different analysis methods and the one-vs-all approach.

	Class 1	Class 2	Number of variables	Settings	Individuals correct prior to exclusion	Individuals incorrect prior to exclusion	Exclusion range	Individuals correct after exclusion	Individuals incorrect after exclusion	Nests correct after exclusion
**PLS**	*T. alpestre*	*T. caespitum*/*impurum*/sp. B	1,801	12	26 (57.8%)	19 (42.2%)	1.25–1.75	21 (46.7%)	0 (0.0%)	10 (66.7%)
	*T. caespitum*	*T. alpestre*/*impurum*/sp. B	1,801	10	20 (43.4%)	25 (56.6%)	1.20–1.80	3 (6.7%)	0 (0.0%)	3 (20.0%)
	*T. impurum*	*T. alpestre*/*caespitum*/sp. B	1,801	15	20 (43.4%)	25 (56.6%)	1.20–1.80	8 (17.8%)	0 (0.0%)	7 (46.7%)
	*T*. sp. B	*T. alpestre*/*caespitum*/*impurum*	1,801	10	9 (20.0%)	36 (80.0%)	1.35–1.65	2 (4.4%)	0 (0.0%)	2 (13.3%)
				**Total**	**75 (41.7%)**	**105 (58.3%)**		**34 (18.9%)**	**0 (0.0%)**	**22 (36.7%)**
**ANN**	*T. alpestre*	*T. caespitum*/*impurum*/sp. B	150	5	35 (77.8%)	10 (22.2%)	–	0 (0.0%)	0 (0.0%)	0 (0.0%)
	*T. caespitum*	*T. alpestre*/*impurum*/sp. B	150	23	19 (42.2%)	26 (57.8%)	–	0 (0.0%)	0 (0.0%)	0 (0.0%)
	*T. impurum*	*T. alpestre*/*caespitum*/sp. B	150	34	25 (55.6%)	20 (44.4%)	–	0 (0.0%)	0 (0.0%)	0 (0.0%)
	*T*. sp. B	*T. alpestre*/*caespitum*/*impurum*	150	18	24 (53.3%)	21 (46.7%)	–	0 (0.0%)	0 (0.0%)	0 (0.0%)
				**Total**	**103 (57.2%)**	**77 (42.8%)**		**0 (0.0%)**	**0 (0.0%)**	**0 (0.0%)**
**RF**	*T. alpestre*	*T. caespitum*/*impurum*/sp. B	150	12	3 (6.7%)	42 (93.3%)	0.35–0.65	2 (4.4%)	0 (0.0%)	2 (13.3%)
	*T. caespitum*	*T. alpestre*/*impurum*/sp. B	150	12	4 (8.9%)	41 (91.1%)	0.10–0.90	0 (0.0%)	0 (0.0%)	0 (0.0%)
	*T. impurum*	*T. alpestre*/*caespitum*/sp. B	150	12	3 (6.7%)	42 (93.3%)	–	0 (0.0%)	0 (0.0%)	0 (0.0%)
	*T*. sp. B	*T. alpestre*/*caespitum*/*impurum*	150	12	4 (8.9%)	41 (91.1%)	0.20–0.80	0 (0.0%)	0 (0.0%)	0 (0.0%)
				**Total**	**14 (7.8%)**	**166 (92.2%)**		**2 (1.1%)**	**0 (0.0%)**	**2 (3.3%)**
	*T. alpestre*	*T. caespitum*/*impurum*/sp. B	1,801	42	2 (4.4%)	43 (95.6%)	0.30–0.70	2 (4.4%)	0 (0.0%)	2 (13.3%)
	*T. caespitum*	*T. alpestre*/*impurum*/sp. B	1,801	42	13 (28.9%)	32 (71.1%)	0.15–0.85	2 (4.4%)	0 (0.0%)	2 (13.3%)
	*T. impurum*	*T. alpestre*/*caespitum*/sp. B	1,801	42	14 (31.1%)	31 (68.9%)	–	0 (0.0%)	0 (0.0%)	0 (0.0%)
	*T*. sp. B	*T. alpestre*/*caespitum*/*impurum*	1,801	42	9 (20.0%)	36 (80.0%)	0.25–0.75	2 (4.4%)	0 (0.0%)	2 (13.3%)
				**Total**	**38 (21.1%)**	**142 (78.9%)**		**6 (3.3%)**	**0 (0.0%)**	**6 (10.0%)**

**Notes.**

PLS Partial least squares regression ANN Artificial neural networks RF Random forests Settings Number of factors (for PLS), number of hidden neurons (for ANN), and optimum mtry, i.e., number of variables used for searching the best split at each node (for RF)

– Indicates that the model never reached the 100% correct classification for the Class 2 validation-set individuals at any range of prediction values.

ANN never resulted in the correct classification of all Class 2 validation-set specimens (Table 2). Unambiguous identification of specimens thus was not possible.

RF identified two individuals as *T. alpestre* (4.4%) using 150 variables, and two individuals (4.4%) each as *T. alpestre*, *T. caespitum*, and *T*. sp. B using 1,801 variables, but none of the workers of *T. impurum* using either variable number. The inclusion of nestmates resulted in an identification success of 13.3% (*T. alpestre*, *T. caespitum, T.* sp. B) and a total of six identified nests (18 individuals, 10.0%) using 1,801 variables. The reduction of variables to 150 resulted in two identified nests (6 individuals, 3.3%).

## Discussion

We showed that an unsupervised method (PCA) was not suitable for pattern recognition in our fibre-optic NIRS data, necessitating supervised approaches such as PLS (Fig. 5). Referring to the major questions of this study, we demonstrated that fibre-optic NIRS can be used as a fast pre-screening method for the unambiguous identification of more than two cryptic ant species despite their morphological and ecological similarities. We also showed that with our implementation of data analysis, PLS is most efficient in predicting the correct species, followed by RF and ANN. Moreover, we demonstrated that the one-vs-all strategy is the only practical possibility of multi-class reduction.

The choice of the most appropriate technique to reduce the multi-class system to a two-class system, as is necessary for PLS, is crucial for the time management of a project. The differences of time expenditure across the three strategies tested increased with increasing number of classes to be investigated (Fig. 6). Considering our ultimate intention to apply the NIRS routine not just to the four species, but to evaluate the applicability of a NIRS-based identification routine to any conceivable multi-class system, the one-vs-all strategy was the only acceptable option. We suggest it as the general approach to reducing multi-class to two-class systems for NIRS identification purposes.

NIRS identification using PLS was able to identify 66.7% of *T. alpestre* and 46.7% of *T. impurum* workers (Table 2) without uncertainty but was less efficient for the discrimination of *T. caespitum* and *T*. sp. B with 20.0% and 13.3% of workers unambiguously identified, respectively. This may be explicable by the high similarity of the CHC profiles of *T. caespitum* and *T*. sp. B, whereas *T. impurum* differs in its CHC profile from all other species (Schlick-Steiner et al., 2006). The PLS regression coefficients showed a few wavelength regions important for species classification that can be found in all four PLS models (for examples, see Fig. S1). Several other regions were shared only by two or three models, or are unique to a model underlining the possible differentiation of the species. Some of these regions were either in a visible range, indicating colour differences, or correspond to CH_2_ and CH_3_ first, second, and combination overtones (Shenk, Workman & Westerhaus, 2008). CH_2_ and CH_3_ are part of insect cuticular hydrocarbons and other lipids (Lockey, 1988) and are important for species recognition (Blomquist & Bagnères, 2010). The analysis of the cuticular hydrocarbon profile of *T. alpestre* using gas chromatography-mass spectrometry and a critical comparison with the profiles of the other *Tetramorium* species has not been done to date and would be required for a final assessment.

ANN were not able to identify any specimen with 100% certainty (Table 2), at least in our implementation of the analysis. In contrast, two other studies that used NIRS showed high success for species identification using ANN: Aldrich et al. (2007) recommended ANN as preferable tool, which performed better than PLS in their study on the NIRS identification of termite species and subspecies, and Dowell et al. (1999) reported both ANN and PLS as very efficient methods for the identification of different genera or groups of species. However, none of the studies investigated a group of cryptic species. The limited ability of ANN to handle efficiently data sets with many variables and few observations is a major disadvantage in comparison to PLS and RF (Svetnik et al., 2003; Liu et al., 2013) and may provide a reasonable explanation for the unsatisfactory results in our study. Similar to Aldrich et al. (2007), we selected 150 variables by stepwise increments of 12 nm, and it is possible that spectral regions important for species discrimination were lost by chance in this process. As the aim of our study was to evaluate the classification performance of each method separately and without intensive pre-processing, we did not select regions based on the PLS regression coefficients to increase the identification success of the ANN analysis. However, it may be possible indeed that this approach would increase the identification success of ANN.

Until now, RF have rarely been used for classification of NIRS data, but Lee et al. (2012) were able to discriminate agricultural products of different geographical origin with up to 100% accuracy. In contrast, our maximum identification success was 13.3% in *T. alpestre*, *T. caespitum*, and *T*. sp. B workers, while none of *T. impurum* were identified in our implementation of the analysis (Table 2). Neither altered variable numbers nor higher numbers of trees (ntree) nor altered numbers of variables used for searching the best split at each node (mtry) increased the number of identified specimens using RF (data not shown). Menze et al. (2009) evaluated the performance of RF and other chemometric methods on NIRS data and concluded to use not just one method but to combine RF for feature selection with PLS for classification. We did not follow this approach because our goal was to evaluate each analysis method separately. Thus, we conclude that raw NIRS data of the *Tetramorium* species investigated in this study, and possibly for other cryptic species, are not suitable for classification analysis using RF.

Compared with other studies, the number of misclassifications by PLS when no prediction values were excluded was high (42.2–80.0%). Jia et al. (2007) and Aldrich et al. (2007) were able to differentiate species with up to 100% classification success without excluding specimens. Examples for intermediate error rates are Dowell et al. (1999) with up to 45% of some stored-grain species combinations and Mayagaya et al. (2009) with 35% for the classification of gravid mosquitoes. This indicates that the efficiency of NIRS depends, among others, on the taxa under investigation, i.e., that not all species can be discriminated with the same classification success. This may explain why the four cryptic species of the *Tetramorium caespitum*/*impurum* complex used in our study were not identified with higher success prior to the exclusion of prediction values. Nevertheless, crypsis is an anthropocentric point of view, and many animals recognise conspecifics by other than visual signals (Bickford et al., 2007). Recognition by chemical profiles is widespread among insects and probably more important than other signals (Blomquist & Bagnères, 2010). Consequently, surface-chemical discrimination of morphologically difficult species is often simple (Seppä et al., 2011; Berville et al., 2013), stressing the relevance of NIRS in species identification. Klarica et al. (2011) assumed interspecific hybridisation as one possible reason for the misidentification of two *T. impurum* samples using NIRS data. However, given the lack of conflict between the mtDNA-based and the morphometrics-based identification results, we do not expect hybridisation in the current data set. Therefore, the high misclassification rate prior to the exclusion of prediction values cannot be explained by hybridisation, even though hybridisation cannot be ruled out ultimately.

To bring the identification method as close as possible to common practice, all specimens used in this study were stored in absolute ethanol before mounting. Aldrich et al. (2007) successfully identified termite species and subspecies using ethanol-preserved specimens, and Rodriguez-Fernandez et al. (2011) identified several fly species. Furthermore, Klarica et al. (2011) used ethanol-stored specimens for *Tetramorium* identification with near-infrared imaging spectroscopy, and Perez-Mendoza et al. (2002) predicted the age of ethanol-stored specimens with similar success as when using fresh ones. It is true that Dowell, Noutcha & Michel (2011) showed in their comparison of different storage media for mosquito age prediction that various media are better than ethanol in their prediction performance. However, our aim was to test whether working with a very widely used (even if suboptimal for NIRS data generation) killing and preserving agent would still allow for correct species identification. Our results suggest that when excluding specimens with intermediate prediction values, satisfying identification results can be achieved. Moreover, no clustering of specimens with identical ethanol storage periods was detectable in a PCA, indicating little influence of the time spent in ethanol (Fig. S2).

To achieve unambiguous identifications, i.e., a residual risk of misidentification of zero within the available data, with our NIRS routine, the exclusion of all false-positive results is necessary. In doing so, individuals in a particular, ambiguous prediction-value range need to be excluded and thus remain unidentified. The pool of excluded individuals can also contain correctly identified individuals if their prediction values fall into the ambiguous range. This loss of individuals is the most distressing cost of achieving unambiguous classification. Based on our results, we go beyond the approach of just excluding ambiguous prediction values and additionally suggest the one-vs-all strategy as a novel standard for the analysis of groups of more than two species using NIRS. Multi-class problems are not limited to complexes of cryptic species but are frequent in biology and other disciplines, e.g., in the classification of age cohorts, crop pests, or food origin (Dowell et al., 1999; Aw, Dowell & Ballard, 2012; Liu et al., 2013). We showed that the approach presented here is a useful pre-screening identification tool for a group of cryptic ant species. Moreover, it may be suitable for any kind of multi-class problem where NIRS can be applied including ones for which, to our knowledge, NIRS has not been applied to date, such as identifying the geographic origin of invasive species or the infection status of target organisms when multiple pathogen strains are involved.

Fibre-optic NIRS is, with a measurement time of one minute per specimen, much faster than other methods. The whole procedure for the identification of 96 specimens takes in total 2.3 h of turn-around time including 1.8 h of hands-on time using our NIRS routine with PLS and the one-vs-all strategy, 65.6 h of turn-around and hands-on time using the morphometric character set established by Steiner et al. (2010), and 23.2 h of turn-around time including 13.3 h of hands-on time using mtDNA for genetic identification as applied in this study (Table S3). This time efficiency, the low running costs, and the absence of lab consumables, sometimes including toxic components, make fibre-optic NIRS an attractive identification tool.

## Conclusions

Fibre-optic NIRS in combination with PLS proved to be an appropriate tool for the unambiguous identification of data on cryptic ant species, albeit at the cost of excluding specimens from identification. Furthermore, the one-vs-all strategy turned out to be a very handy possibility to study groups containing more than two species. We conclude that our NIRS classification routine provides a fast and inexpensive tool for multi-class species identification.

## Supplemental Information

10.7717/peerj.991/supp-1 Table S1 Information on the *Tetramorium* ant sample Species identity, geographic location of nest, date of collection, collector, and sample code.

10.7717/peerj.991/supp-2 Table S2 Results of the species classification for artificial neural networks (ANN) and random forests (RF) without reducing the four-class system to a two-class system

10.7717/peerj.991/supp-3 Table S3 Time estimation for *Tetramorium* species identification Estimated time for the identification of 96 individuals using near-infrared spectroscopy (NIRS), morphometrics, and molecular genetics (mtDNA) assuming that optimum partial least squares regression models, the routine identification tool for morphological characteristics (http://web-resources.boku.ac.at/Discmean/), and reference mtDNA sequences in GenBank are available. Hands-on time was determined as active working time and turn-around time as total working time including waiting times (e.g., incubation).

10.7717/peerj.991/supp-4 Figure S1 Regression coefficients of the partial least squares regression for the classification of the four *Tetramorium* species using the one-vs-all strategy (A) *Tetramorium alpestre* versus *T. caespitum* / *impurum*/ sp. B, (B) *T. caespitum* versus *T. alpestre* / *impurum*/ sp. B, (C) *T. impurum* versus *T. alpestre* / *caespitum*/ sp. B, and (D) *T.* sp. B versus *T. alpestre* / *caespitum* / *impurum*. Vertical broken lines show examples of wavelengths relevant to identifying one, two, three, or four species as indicated by the coloured circles at the top of the graph (red = *T. alpestre*, green = *T. caespitum*, yellow = *T. impurum*, and purple = *T.* sp. B).

10.7717/peerj.991/supp-5 Figure S2 Principal component analysis scatter plot of 1,801 spectral variables from all 528 specimens for ethanol (EtOH) storage periods Specimens with identical ethanol storage period share the same colour. Percentage of the variation explained by each of the first two principal components (PC) given. There is no indication of clustering.

10.7717/peerj.991/supp-6 Data S1 NIRS data for all *Tetramorium* specimens used in this study

## Additional Information and Declarations

### DNA Deposition

The following information was supplied regarding the deposition of DNA sequences: New sequences have been submitted to GenBank under the accession numbers KT248392–KT248508.
