# Supplementary material for: A near-infrared spectroscopy routine for unambiguous identification of cryptic ant species
Source: PeerJ. 2015 Sep 15;3:e991. doi: 10.7717/peerj.991 (PMC4699785; doi:10.7717/peerj.991)
Supplement: Table S1 — Species identity, geographic location of nest, date of collection, collector, and sample code. [file peerj-03-991-s001.doc]

| **Species** | **Locality** | **Lon** | **Lat** | **Alt** | **Date** | **Collector** | **Sample code** |
| --- | --- | --- | --- | --- | --- | --- | --- |
| ***T. alpestre*** | AU: Dobratsch | 13.71 | 46.59 | 1730 | 21.VII.2002 | BSS, FMS | TM280 |
| ***T. alpestre*** | AU: Haggen | 11.08 | 47.22 | 1800 | 15.VI.2010 | LR | 17752 |
| ***T. alpestre*** | AU: Haggen | 11.08 | 47.21 | 1680 | 25.VI.2010 | LR | 17758 |
| ***T. alpestre*** | AU: Haggen | 11.08 | 47.21 | 1800 | 12.VII.2010 | LR | 17793 |
| ***T. alpestre*** | AU: Haggen | 11.08 | 47.21 | 1800 | 12.VII.2010 | LR | 17795 |
| ***T. alpestre*** | AU: Haggen | 11.10 | 47.21 | 1947 | 14.VII.2010 | LR | 17798 |
| ***T. alpestre*** | AU: Kühtai | 10.99 | 47.22 | 1760 | 25.VI.2010 | LR | 17757 |
| ***T. alpestre*** | AU: Kühtai | 10.99 | 47.22 | 1760 | 30.VI.2010 | LR | 17764 |
| ***T. alpestre*** | AU: Obergurgl | 11.02 | 46.87 | 1900 | 19.VII.2009 | HZ | 17666 |
| ***T. alpestre*** | AU: Obergurgl | 11.02 | 46.87 | 1900 | 07.VIII.2001 | BSS, FMS | TM100 |
| ***T. alpestre*** | AU: Obergurgl | 11.02 | 46.87 | 1900 | 07.VIII.2001 | BSS, FMS | TM98 |
| ***T. alpestre*** | AU: Padasterjoch | 11.38 | 47.09 | 1778 | 08.VII.2009 | BSS, FMS | 16865 |
| ***T. alpestre*** | AU: Strajach | 12.92 | 46.70 | 1700 | 19.VII.2002 | BSS, FMS | TM281 |
| ***T. alpestre*** | AU: Strajach | 12.92 | 46.70 | 1700 | 18.VI.2001 | BSS, FMS | TM94 |
| ***T. alpestre*** | AU: Strajach | 12.92 | 46.70 | 1700 | 18.VI.2001 | BSS, FMS | TM96 |
| ***T. alpestre*** | FR: Ailefroide | 6.40 | 44.88 | 2400 | 03.VIII.2009 | CL | 17020 |
| ***T. alpestre*** | FR: Briancon | 6.65 | 44.88 | 2200 | 28.VII.1994 | TA, RG | i542 |
| ***T. alpestre*** | FR: Jausier | 6.72 | 44.42 | 1300 | 29.VII.1994 | TA, RG | i540 |
| ***T. alpestre*** | IT: Hütt | 11.13 | 46.82 | 1480 | 07.VIII.2007 | BS | 16720 |
| ***T. alpestre*** | IT: Hütt | 11.13 | 46.82 | 1480 | 07.VIII.2007 | BS | 16722 |
| ***T. alpestre*** | IT: Jaufenpass | 11.25 | 46.82 | 1900 | 01.VIII.2000 | AB | i46 |
| ***T. alpestre*** | IT: Molise | 13.95 | 41.69 | 1778 | 09.VII.2009 | MM | 17081 |
| ***T. alpestre*** | IT: Monte Baldo | 10.83 | 45.73 | 1450 | 27.VII.2002 | AB | i611 |
| ***T. alpestre*** | IT: Penser Joch | 11.45 | 46.83 | 2043 | 24.VI.2010 | LR | 17756 |
| ***T. alpestre*** | IT: Penser Joch | 11.45 | 46.83 | 2015 | 06.VII.2010 | LR | 17770 |
| ***T. alpestre*** | IT: Penser Joch | 11.44 | 46.83 | 2048 | 08.VII.2010 | LR | 17771 |
| ***T. alpestre*** | IT: Penser Joch | 11.44 | 46.83 | 2040 | 08.VII.2010 | LR | 17772 |
| ***T. alpestre*** | IT: Pfelders | 11.07 | 46.80 | 2160 | 06.VIII.2007 | BS | 16701 |
| ***T. alpestre*** | IT: Pfelders | 11.06 | 46.78 | 1800 | 26.VI.2010 | JK | 17760 |
| ***T. alpestre*** | IT: Pfelders | 11.06 | 46.78 | 1800 | 26.VI.2010 | JK | 17761 |
| ***T. alpestre*** | IT: Pfelders | 11.06 | 46.78 | 1830 | 26.VI.2010 | JK | 17762 |
| ***T. alpestre*** | IT: Piz Lad | 10.49 | 46.83 | 2100 | 09.VII.2009 | BSS, FMS | 16868 |
| ***T. alpestre*** | IT: Plampincieux | 7.00 | 45.83 | 1762 | 9.-13.VI.09 | WM | 17529 |
| ***T. alpestre*** | SZ: Alp La Schera | 10.20 | 46.64 | 2094 | 09.VIII.2007 | BS | 16726 |
| ***T. alpestre*** | SZ: Champlönch | 10.17 | 46.68 | 1845 | 17.VIII.2007 | AF, DC | 16739 |
| ***T. alpestre*** | SZ: Engiloch | 8.02 | 46.22 | 1806 | 03.X.2009 | HMA | 17437 |
| ***T. alpestre*** | SZ: Engiloch | 8.02 | 46.22 | 1803 | 03.X.2009 | HMA | 17439 |
| ***T. alpestre*** | SZ: Lago di Robiei | 8.51 | 46.44 | 1920 | 12.VIII.2007 | RN | 16728 |
| ***T. alpestre*** | SZ: Paltano | 8.44 | 46.47 | 1918 | 10.IX.2007 | RN | 16743 |
| ***T. alpestre*** | SZ: S-Chanf | 9.98 | 46.61 | 1700 | 8.VII.2000 | PD | i70 |
| ***T. alpestre*** | SZ: Scuol | 10.26 | 46.82 | 2335 | 01.VII.2007 | RN | 16702 |
| ***T. caespitum*** | AU: Innsbruck | 11.38 | 47.27 | 720 | 07.VIII.2009 | JK | 16976 |
| ***T. caespitum*** | AU: Perchtoldsdorf | 16.23 | 48.12 | 400 | 30.VII.2000 | BSS, FMS | TM40 |
| ***T. caespitum*** | GM: Ingelheim | 8.07 | 49.97 | 130 | 30.VI.2009 | GH | 17006 |
| ***T. caespitum*** | AU: vic. Schwarz | 14.39 | 46.58 | 760 | 17.VI.2003 | BSS, FMS | TM363 |
| ***T. caespitum*** | AU: Wels | 14.03 | 48.18 | 320 | 13.VII.2009 | JA | 17323 |
| ***T. caespitum*** | AU: Zwaring-Pöls | 15.44 | 46.90 | 315 | 13.VI.2009 | HCW | 17447 |
| ***T. caespitum*** | BE: Peer | 5.48 | 51.06 | 76 | 05.VIII.2009 | FV | 17696 |
| ***T. caespitum*** | BE: Zonhoven | 5.40 | 51.01 | 61 | 30.VIII.2009 | FV | 17698 |
| ***T. caespitum*** | DA: Tuno Island | 10.42 | 55.95 | 30 | 8.VI.2000 | MN | i11 |
| ***T. caespitum*** | EN: Alliku | 27.27 | 59.07 | 63 | 12.VIII.2009 | JM, TM | 17401 |
| ***T. caespitum*** | EN: Saremaa | 22.50 | 58.42 | 30 | 12.VII.2000 | KV | i71 |
| ***T. caespitum*** | EZ: Chudenice | 13.19 | 49.47 | 510 | 30.VIII.2009 | PB, KB | 17554 |
| ***T. caespitum*** | EZ: Citoliby | 13.83 | 50.33 | 350 | 31.VIII.2009 | PP | 17704 |
| ***T. caespitum*** | EZ: Drmaly | 13.46 | 50.52 | 340 | 04.VIII.2009 | MH | 17000 |
| ***T. caespitum*** | EZ: Holubov | 14.32 | 48.87 | 600 | 18.VII.2000 | JF | i63 |
| ***T. caespitum*** | EZ: Rana | 13.77 | 50.40 | 413 | 04.VII.2009 | PW | 17362 |
| ***T. caespitum*** | EZ: Zbelitov | 14.31 | 49.46 | 490 | 30.VIII.2009 | PB, KB | 17556 |
| ***T. caespitum*** | FI: Tvärminne | 23.20 | 59.83 | 8 | 2000 | LSU | i20 |
| ***T. caespitum*** | GM: Annweiler am Trifels | 7.96 | 49.20 | 362 | 14.-17.VI.2009 | GH | 16845 |
| ***T. caespitum*** | GM: Babenhausen | 8.95 | 49.97 | 132 | 06.VII.2000 | AB | i16 |
| ***T. caespitum*** | GM: Cochem an der Mosel | 7.15 | 50.12 | 330 | 14.VII.1995 | MS | i679 |
| ***T. caespitum*** | AU: Innsbruck | 11.36 | 47.27 | 606 | 14.VI.2009 | JK | 16836 |
| ***T. caespitum*** | GM: Prittrichinger Heide | 10.92 | 48.20 | 540 | VII.2000 | PH | i52 |
| ***T. caespitum*** | GM: Würzburg | 9.93 | 49.79 | 300 | 28.V.2009 | FM | 16974 |
| ***T. caespitum*** | GM: Zeilhard | 8.79 | 49.84 | 200 | 17.VI.2009 | AB | 16993 |
| ***T. caespitum*** | HR: Fazana | 13.80 | 44.92 | 2 | 17.VIII.2009 | HCW | 17461 |
| ***T. caespitum*** | HU: Bács-Kiskun | 19.50 | 46.60 | 110 | 5.IX.1993 | MS | i285 |
| ***T. caespitum*** | IT: Latsch | 10.85 | 46.62 | 800 | 29.VIII.2009 | HM | 17504 |
| ***T. caespitum*** | IT: Pescasseroli | 13.79 | 41.81 | 1400 | 08.VII.2009 | MM | 17080 |
| ***T. caespitum*** | IT: Pra Bestema | 10.76 | 45.65 | 850 | 27.VI.2009 | ASP | 17289 |
| ***T. caespitum*** | SZ: Schaffhausen | 8.60 | 47.79 | 650 | 11.VIII.2009 | RN | 17165 |
| ***T. caespitum*** | LS: Ruggell | 9.52 | 47.25 | 445 | 27.VIII.2009 | FG | 17653 |
| ***T. caespitum*** | NL: Reusel | 5.17 | 51.37 | 30 | 22.VII.2000 | YR | i41 |
| ***T. caespitum*** | PL: Kampinos National Park | 20.46 | 52.27 | 75 | 24.VII.2000 | JPE | i36 |
| ***T. caespitum*** | PL: Strupina | 16.80 | 51.38 | 170 | 28.VI.2009 | MBO | 17416 |
| ***T. caespitum*** | SW: Floghult Bohuslan | 11.42 | 58.97 | 100 | 21.VI.2000 | CC | i132 |
| ***T. caespitum*** | SW: Kyrkheddinge | 13.27 | 55.67 | 100 | 2.-10.IX.2009 | PDO | 17088 |
| ***T. caespitum*** | SZ: Cadepezzo | 8.90 | 46.16 | 217 | 06.IV.2009 | CB | 17492 |
| ***T. caespitum*** | SZ: Ligerz | 7.15 | 47.09 | 520 | 10.IX.2009 | RN | 17168 |
| ***T. caespitum*** | SZ: Lottigna | 8.93 | 46.47 | 560 | 7.VIII.2000 | RN | i60 |
| ***T. caespitum*** | AU: Ötz | 10.90 | 47.21 | 900 | 08.VI.2007 | HM | 16747 |
| ***T. caespitum*** | SZ: Wettswil am Albis | 8.46 | 47.34 | 530 | 09.VIII.2010 | RN | 17849 |
| ***T. caespitum*** | UK: Deepcut | -0.70 | 51.28 | 75 | 8.VI.2000 | JP | i1 |
| ***T. caespitum*** | UK: Isle of Purbeck | -2.02 | 50.70 | 45 | VIII.2000 | JW | i53 |
| ***T. caespitum*** | UK: Whitcombe Vale | -2.38 | 50.67 | 140 | VIII.2000 | JW | i56 |
| ***T. impurum*** | AU: Haggen | 11.08 | 47.21 | 1680 | 30.VI.2010 | LR | 17765 |
| ***T. impurum*** | AU: Hochpillberg | 11.72 | 47.32 | 1400 | 31.V.2009 | HM | 17496 |
| ***T. impurum*** | AU: Hofstätten | 16.10 | 47.98 | 430 | 14.X.2001 | BSS, FMS | TM104 |
| ***T. impurum*** | AU: Hollenstein vic. Scheibbs | 15.27 | 47.96 | 650 | 03.VIII.2002 | BSS, FMS | TM308 |
| ***T. impurum*** | AU: Innsbruck | 11.36 | 47.27 | 604 | 14.VII.2009 | JK | 16837 |
| ***T. impurum*** | AU: Lassingbach | 15.08 | 47.73 | 740 | 09.VI.2001 | BSS, FMS | TM91 |
| ***T. impurum*** | AU: Losenheim | 15.82 | 47.78 | 870 | 31.V.2001 | BSS, FMS | TM89 |
| ***T. impurum*** | AU: Scharnitz | 11.27 | 47.39 | 1176 | 04.X.2012 | MG | 18223 |
| ***T. impurum*** | AU: Schildbachgraben | 15.49 | 48.42 | 420 | 03.IX.2003 | BSS, FMS | TM402 |
| ***T. impurum*** | AU: Terfens | 11.66 | 47.33 | 701 | 24.IX.2012 | HCW | 18218 |
| ***T. impurum*** | AU: Weissenbach | 15.27 | 48.02 | 700 | 03.VIII.2002 | BSS, FMS | TM312 |
| ***T. impurum*** | BE: Ampsin | 5.28 | 50.53 | 84 | 28.VII.2009 | PWG | 17051 |
| ***T. impurum*** | BE: Diest | 5.04 | 50.99 | 22 | 04.VIII.2009 | FV | 17694 |
| ***T. impurum*** | BE: Hainaut | 4.30 | 50.35 | 153 | 26.VIII.2009 | PWG | 17387 |
| ***T. impurum*** | BE: Liège | 5.60 | 50.60 | 275 | 31.VIII.2009 | PWG | 17060 |
| ***T. impurum*** | BE: Mirwart | 5.27 | 50.03 | 360 | 4.VIII.2000 | YR | i73 |
| ***T. impurum*** | BE: Namur | 4.59 | 50.19 | 290 | 22.VIII.2009 | PWG | 17382 |
| ***T. impurum*** | BH: Bjelasnica | 18.31 | 43.70 | 1254 | 22.VIII.2011 | AV | 18017 |
| ***T. impurum*** | BU: Karandila | 26.35 | 42.72 | 1050 | 22.VII.2009 | TL | 16957 |
| ***T. impurum*** | EZ: Hacava | 20.82 | 48.67 | 650 | 29.VII.2002 | AT | i668 |
| ***T. impurum*** | EZ: Holubov | 14.32 | 48.87 | 600 | 18.VII.2000 | JF | i62 |
| ***T. impurum*** | EZ: Kamyk-Plesivec | 14.08 | 50.55 | 360 | 05.VII.2009 | PP | 17706 |
| ***T. impurum*** | EZ: Pitarne | 17.59 | 50.25 | 350 | 26.VIII.2009 | PB, KB | 17553 |
| ***T. impurum*** | FR: Le Bois-Plage-en-Ré | 1.39 | 46.19 | 7 | 27.IX.2009 | CG | 17300 |
| ***T. impurum*** | FR: Le Porge | -1.21 | 44.89 | 11 | 17.V.2009 | CG | 16815 |
| ***T. impurum*** | GM: Annweiler am Trifels | 7.97 | 49.20 | 360 | 12.IX.2000 | GH | i76 |
| ***T. impurum*** | AU: Stubai | 11.31 | 47.12 | 1511 | 24.VII.2009 | JK | 16937 |
| ***T. impurum*** | GM: Geisingen | 8.67 | 47.92 | 637 | 25.IX.2009 | HMA | 17432 |
| ***T. impurum*** | GM: Spitzingsee | 11.91 | 47.67 | 1400 | VI.2007 | GH | 16714 |
| ***T. impurum*** | HU: Komarom | 18.13 | 47.74 | 200 | 01.IX.1993 | MS | i594 |
| ***T. impurum*** | AU: Ötz | 10.90 | 47.21 | 900 | 08.VI.2007 | HM | 16748 |
| ***T. impurum*** | LO: Bokšovskà skala | 21.12 | 48.87 | 600 | 24.VII.2009 | MSU | 17146 |
| ***T. impurum*** | LS: Balzers | 9.50 | 47.06 | 477 | 13.IX.2009 | HMA | 17217 |
| ***T. impurum*** | LS: Balzers | 9.48 | 47.07 | 490 | 25.VIII.2009 | FG | 17657 |
| ***T. impurum*** | LS: Bargälla | 9.55 | 47.13 | 1580 | 26.VIII.2009 | FG, HF | 17654 |
| ***T. impurum*** | MJ: Zabljak | 19.13 | 43.17 | 1400 | 16.VIII.2009 | MB | 17686 |
| ***T. impurum*** | PL: Słupice | 16.74 | 50.83 | 360 | 16.VII.2009 | MBO | 17425 |
| ***T. impurum*** | SP: Javalambre Teruel | -1.00 | 40.10 | 2000 | 7.VII.2000 | CC | i126 |
| ***T. impurum*** | SP: Punta Peña | -3.28 | 43.36 | 400 | 15.VII.2010 | JG | 17855 |
| ***T. impurum*** | SZ: Chalet a Roch | 6.18 | 46.54 | 1430 | VI.2007 | CB, DC | 16734 |
| ***T. impurum*** | SZ: Chandonne | 7.18 | 46.00 | 1210 | 10.IX.2009 | HMA | 17191 |
| ***T. impurum*** | SZ: Guarda | 10.15 | 46.77 | 1470 | 29.VI.2007 | RN | 16712 |
| ***T. impurum*** | SZ: La St. George | 6.27 | 46.53 | 1180 | 12.IX.2009 | AF | 17257 |
| ***T. impurum*** | SZ: Nossplatten | 8.75 | 46.78 | 1715 | 24.VI.2007 | RN | 16703 |
| ***T. impurum*** | SZ: Tete de Ran | 6.85 | 47.05 | 1365 | 25.VIII.2007 | AF | 16737 |
| ***T.* sp. B** | AM: Blagodarnoye | 44.02 | 41.07 | 1950 | 1.VII.2002 | MW | i653 |
| ***T.* sp. B** | AM: Gjumri | 43.87 | 40.88 | 1900 | 13.VII.2002 | MW | i654 |
| ***T.* sp. B** | AU: Feldberg | 15.67 | 48.85 | 298 | 02.VI.2002 | BSS, FMS | TM261 |
| ***T.* sp. B** | AU: Forchtenstein | 16.33 | 47.70 | 330 | 23.IX.2000 | BSS, FMS | TM63 |
| ***T.* sp. B** | AU: Gnadendorf | 16.40 | 48.62 | 280 | 10.V.2001 | BSS, FMS | TM85 |
| ***T.* sp. B** | AU: Setzberg | 15.40 | 48.36 | 330 | 01.X.2002 | BSS, FMS | TM314 |
| ***T.* sp. B** | AU: Wiener Neustadt | 16.25 | 47.80 | 262 | 05.IX.2000 | BSS, FMS | TM35 |
| ***T.* sp. B** | BH: Janj | 17.15 | 44.20 | 785 | 26.VI.2011 | AV | 17977 |
| ***T.* sp. B** | BH: Kupres | 17.29 | 44.01 | 1232 | 26.VI.2011 | AV | 18096 |
| ***T.* sp. B** | BH: Šavnici | 18.20 | 43.76 | 954 | 10.VII.2011 | AV | 18059 |
| ***T.* sp. B** | BH: Vareš | 18.32 | 44.16 | 925 | 13.VIII.2011 | AV | 18062 |
| ***T.* sp. B** | BU: Bistritsa | 23.36 | 42.59 | 950 | 06.VI.2009 | SC | 18285 |
| ***T.* sp. B** | BU: Parangalitsa reserve | 23.35 | 42.03 | 1470 | 10.VII.2009 | TL | 16959 |
| ***T.* sp. B** | BU: Patreshko | 24.75 | 42.91 | 640 | 09.VIII.2009 | AG | 17483 |
| ***T.* sp. B** | BU: Tihiya Kut | 23.25 | 42.53 | 990 | 19.VI.2002 | CD, TL | i311 |
| ***T.* sp. B** | BU: Tsaparevo | 23.10 | 41.62 | 640 | 25.IX.2002 | TL | i738 |
| ***T.* sp. B** | BU: Vladaya | 23.20 | 42.63 | 900 | 08.VIII.2009 | VA | 17347 |
| ***T.* sp. B** | BU: Vratchansk Mt. | 23.42 | 43.13 | 1260 | 29.IX.2002 | TL | i739 |
| ***T.* sp. B** | EZ: Zašovice | 15.72 | 49.26 | 650 | 26.VI.2009 | PB, KB | 17546 |
| ***T.* sp. B** | GM: Gohrischer Heide | 14.12 | 50.92 | 100 | 8.VIII.1994 | RG, AS | i290 |
| ***T.* sp. B** | GM: Oberleinach | 9.80 | 49.85 | 280 | 18.VII.2000 | UM | i66 |
| ***T.* sp. B** | GR: Killini | 22.42 | 37.95 | 1700 | 5.VI.1994 | AS, KV | i289 |
| ***T.* sp. B** | HR: Tašmarija | 15.28 | 45.31 | 250 | 15.VIII.2009 | JK | 16979 |
| ***T.* sp. B** | HU: Bükkszentkereszt | 20.63 | 48.05 | 600 | 05.VII.2009 | AT | 16928 |
| ***T.* sp. B** | IT: Forgaria nel Friuli | 12.97 | 46.21 | 150 | 27.VIII.2010 | RN | 17851 |
| ***T.* sp. B** | IT: Pra Bestema | 10.76 | 45.65 | 900 | 13.VIII.2009 | ASP | 17291 |
| ***T.* sp. B** | LO: Belá-Dulice | 18.99 | 49.00 | 530 | 21.VII.2009 | WM | 17237 |
| ***T.* sp. B** | LO: Bretka | 20.34 | 48.50 | 195 | 18.VII.2009 | MSU | 17141 |
| ***T.* sp. B** | LO: Kamenná hora | 21.50 | 48.58 | 550 | 17.VII.2009 | MSU | 17139 |
| ***T.* sp. B** | LO: Kozelník | 18.98 | 48.51 | 380 | 21.VI.2009 | WM | 17224 |
| ***T.* sp. B** | LO: Liptovská Lužná | 19.31 | 48.97 | 866 | 30.VII.2009 | MWI | 17244 |
| ***T.* sp. B** | LO: Súdovce | 18.83 | 48.23 | 238 | 09.VII.2009 | MWI | 17230 |
| ***T.* sp. B** | MJ: Sljivansko | 19.30 | 43.13 | 695 | 16.VIII.2009 | MB | 17687 |
| ***T.* sp. B** | RO: Cisnădioara | 24.08 | 45.70 | 550 | 25.VIII.2009 | ITA | 17281 |
| ***T.* sp. B** | RO: Rodan National Park | 24.68 | 47.60 | 1200 | 15.VII.2009 | ITA | 17271 |
| ***T.* sp. B** | SI: Cerknica | 14.51 | 45.78 | 750 | 31.VIII.2002 | GB | i756 |
| ***T.* sp. B** | SI: Grcarice | 14.76 | 45.65 | 528 | 31.VIII.2002 | GB | i757 |
| ***T.* sp. B** | SI: Ljubljana | 14.45 | 46.23 | 300 | 5.VI.2002 | GB | i234 |
| ***T.* sp. B** | SI: Metlika | 15.30 | 45.65 | 200 | 26.VI.2002 | GB | i750 |
| ***T.* sp. B** | SI: Skofja Loka | 14.31 | 46.17 | 350 | 29.V.2002 | GB | i232 |
| ***T.* sp. B** | SI: Vinica | 15.27 | 45.88 | 400 | 26.VI.2002 | GB | i751 |
| ***T.* sp. B** | SI: Vipava | 13.97 | 45.79 | 160 | 10.VI.2002 | GB | i749 |
| ***T.* sp. B** | SI: Zagorica | 15.03 | 45.95 | 380 | 17.V.2009 | GB | 17511 |
| ***T.* sp. B** | UP: Kolachava | 23.68 | 48.43 | 550 | 2000 | AR | i143 |
| ***T.* sp. B** | YI: Dojkinci | 22.78 | 43.23 | 1450 | 06.X.2009 | VV | 17660 |

**Locality.** AM = Armenia, AU = Austria, BE = Belgium, BH = Bosnia-Herzegovina, BU = Bulgaria, DA = Denmark, EN = Estonia, EZ = Czech Republic, FI = Finland, FR = France, GM = Germany, GR = Greece, HR= Croatia, HU = Hungary, IT = Italy, LO = Slovakia, LS = Liechtenstein, MJ = Montenegro, NL = Netherlands, PL = Poland, , RO = Romania, SI = Slovenia, SP = Spain, SW= Sweden, SZ = Switzerland, UK= United Kingdom, UP = Ukraine, YI = Serbia. **Lon.** Longitude in decimal format, positive values indicate position east of Greenwich, negative values indicate position west of Greenwich. **Lat.** Latitude in decimal format. **Alt.** Altitude, m above sea level. **Date.** Date of collection. **Collector.** AB = A. Buschinger, AF = A. Freitag, AG = A. Gjonova, AR = A. Radchenko, AS = A. Schultz, ASP = A. Scupola, AT = A. Tartally, AV = A. Vesnic, BS = B. Seifert, BSS = B. C. Schlick-Steiner, CB = C. Bernasconi, CC = C. Collingwood, CD = C. Deltshev, CG = C. Galkowski, CL = C. Lebas, DC = D. Cherix, FG = F. Glaser, FM = F. Menzel, FMS = F. M. Steiner, FV = F. Vankerkhoven, GB = G. Bracko, GH = G. Heller, HCW = H. C. Wagner, HF = H. Frick, HM = H. Müller, HMA = H. Martz, HZ = H. Zettel, ITA = I. Tausen, JA = J. Ambach, JF = J. Frouz, JG = J. Garcia-Silvares, JK = J. Klarica, JM = J. Martin, JP = J. Pontin, JPE = J. Petal, JW = J. Wardlaw, KB = K. Bezdečkova, KV = K. Vock, LR = L. Russo, LSU = L. Sundström, MB = M. Balint, MBO = M. Borowiec, MG = M. Gassner, MH = M. Holec, MM = M. Mei, MN = M. G. Nielsen, MS = M. Sanetra, MSU = M. Suvak, MW = M. Wuermli, MWI = M. Wiezik, PB = P. Bezdečka, PD = P. Duelli, PDO = P. Douwes, PH = P. Hartmann, PP = P. Pech, PW = P. Werner, PWG = P. Wegnez, RG = R. Günsten, RN = R. Neumeyer, SC = S. Csősz, TA = T. Aßmuth, TL = T. Ljubomirov, TM = T. Martin, UM = U. Maschwitz, VA = V. Antonova, VV = V. Vohralik, WM = M. Witek, YR = Y. Roisin. **Sample code.** Labels of voucher specimens.
