# Supplementary material for: A near-infrared spectroscopy routine for unambiguous identification of cryptic ant species
Source: PeerJ. 2015 Sep 15;3:e991. doi: 10.7717/peerj.991 (PMC4699785; doi:10.7717/peerj.991)
Supplement: Table S2 [file peerj-03-991-s002.doc]

|  | **Species** | **Number of variables** | **Settings** | **Individuals correct** | **Individuals incorrect** |
| --- | --- | --- | --- | --- | --- |
| **ANN** | *T. alpestre* | 150 | 14 | 34 (75.6%) | 11 (24.4%) |
|  | *T. caespitum* | 150 | 14 | 18 (40.0%) | 27 (60.0%) |
|  | *T. impurum* | 150 | 14 | 24 (53.3%) | 21 (46.7%) |
|  | *T.* sp. B | 150 | 14 | 24 (53.3%) | 21 (46.7%) |
|  |  |  | **total** | **100 (55.6%)** | **80 (44.4%)** |
| **RF** | *T. alpestre* | 150 | 12 | 9 (20.0%) | 36 (80.0%) |
|  | *T. caespitum* | 150 | 12 | 25 (55.6%) | 20 (44.4%) |
|  | *T. impurum* | 150 | 12 | 19 (42.2%) | 26 (57.8%) |
|  | *T.* sp. B | 150 | 12 | 15 (33.3%) | 30 (66.7%) |
|  |  |  | **total** | **68 (37.8%)** | **112 (62.2%)** |
|  | *T. alpestre* | 1801 | 42 | 15 (33.3%) | 30 (66.7%) |
|  | *T. caespitum* | 1801 | 42 | 26 (57.8%) | 19 (42.2%) |
|  | *T. impurum* | 1801 | 42 | 24 (53.3%) | 21 (46.7%) |
|  | *T.* sp. B | 1801 | 42 | 21 (46.7%) | 24 (53.3%) |
|  |  |  | **total** | **86 (47.8%)** | **94 (52.2%)** |

**Settings.** Number of hidden neurons (for ANN) and optimum mtry, i.e. number of variables used for searching the best split at each node (for RF).
