# Supplementary material for: A near-infrared spectroscopy routine for unambiguous identification of cryptic ant species
Source: PeerJ. 2015 Sep 15;3:e991. doi: 10.7717/peerj.991 (PMC4699785; doi:10.7717/peerj.991)
Supplement: Table S3 — Estimated time for the identification of 96 individuals using near-infrared spectroscopy (NIRS), morphometrics, and molecular genetics (mtDNA) assuming that optimum partial least squares regression models, the routine identification tool for morphological characteristics (http://web-resources.boku.ac.at/Discmean/), and reference mtDNA sequences in GenBank are available. Hands-on time was determined as active working time and turn-around time as total working time including waiting times (e.g., incubation). [file peerj-03-991-s003.doc]

|  | **Hands-on** | **Turn-around** |
| --- | --- | --- |
| **NIRS** |  |  |
| setup | 5 min | 5 min |
| warming-up Labspec |  | 30 min |
| reference baseline | 1 min | 1 min |
| measurement | 96 min | 96 min |
| prediction | 3 min | 3 min |
| **total** | **1.8 h** | **2.3 h** |
| **Morphometrics** |  |  |
| measurement | 3840 min | 3840 min |
| prediction | 96 min | 96 min |
| **total** | **65.6 h** | **65.6 h** |
| **mtDNA** |  |  |
| DNA extraction | 161 min | 427 min |
| PCR and purification | 96 min | 282 min |
| sequencing | 59 min | 204 min |
| basecalling | 480 min | 480 min |
| **total** | **13.3 h** | **23.2 h** |
