# Supplementary figures and images for: A near-infrared spectroscopy routine for unambiguous identification of cryptic ant species
Source: PeerJ. 2015 Sep 15;3:e991. doi: 10.7717/peerj.991 (PMC4699785; doi:10.7717/peerj.991)

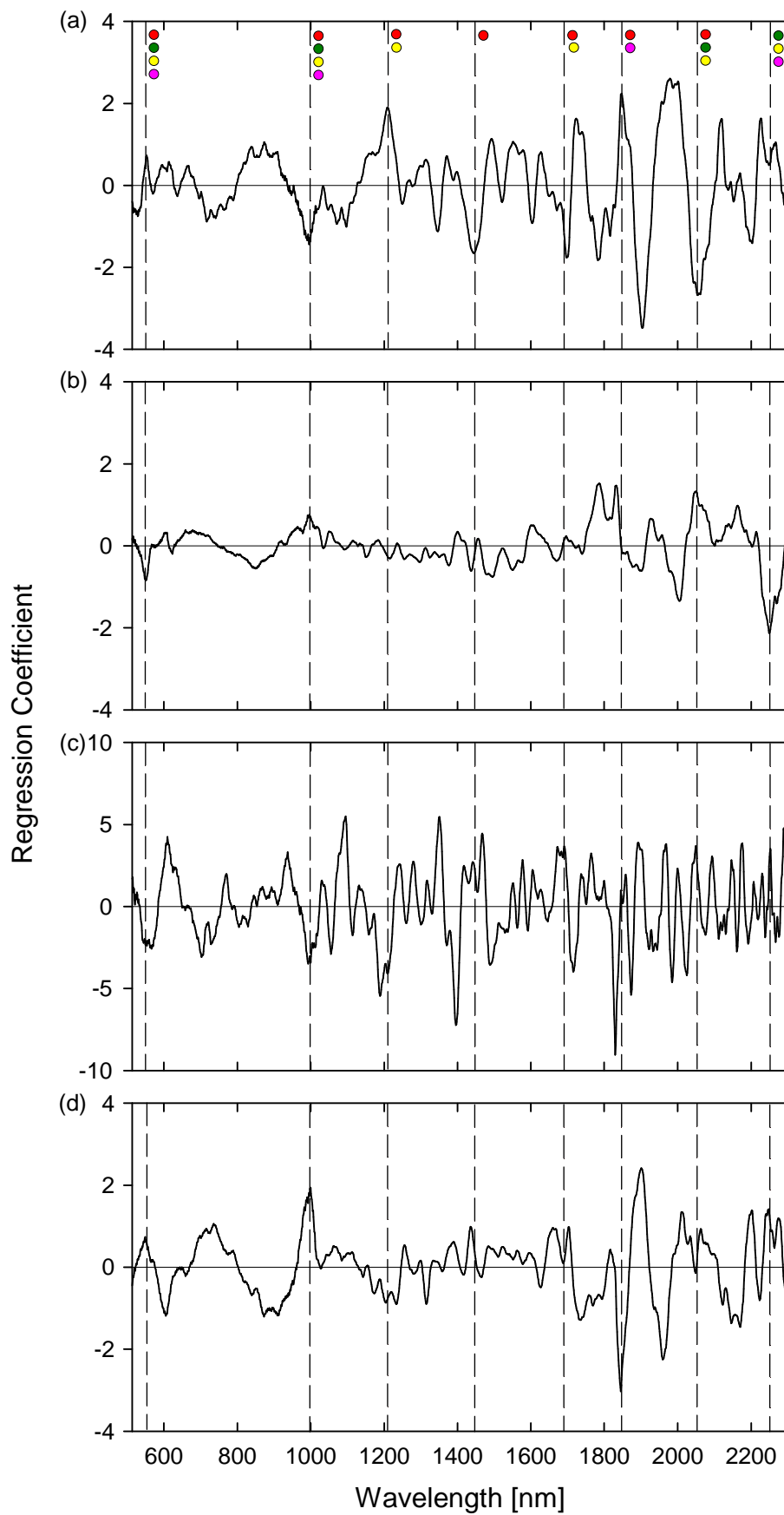

Supplement: Figure S1 — (A) Tetramorium alpestre versus T. caespitum / impurum/ sp. B, (B) T. caespitum versus T. alpestre / impurum/ sp. B, (C) T. impurum versus T. alpestre / caespitum/ sp. B, and (D) T. sp. B versus T. alpestre / caespitum / impurum. Vertical broken lines show examples of wavelengths relevant to identifying one, two, three, or four species as indicated by the coloured circles at the top of the graph (red = T. alpestre, green = T. caespitum, yellow = T. impurum, and purple = T. sp. B). [file peerj-03-991-s004.pdf]

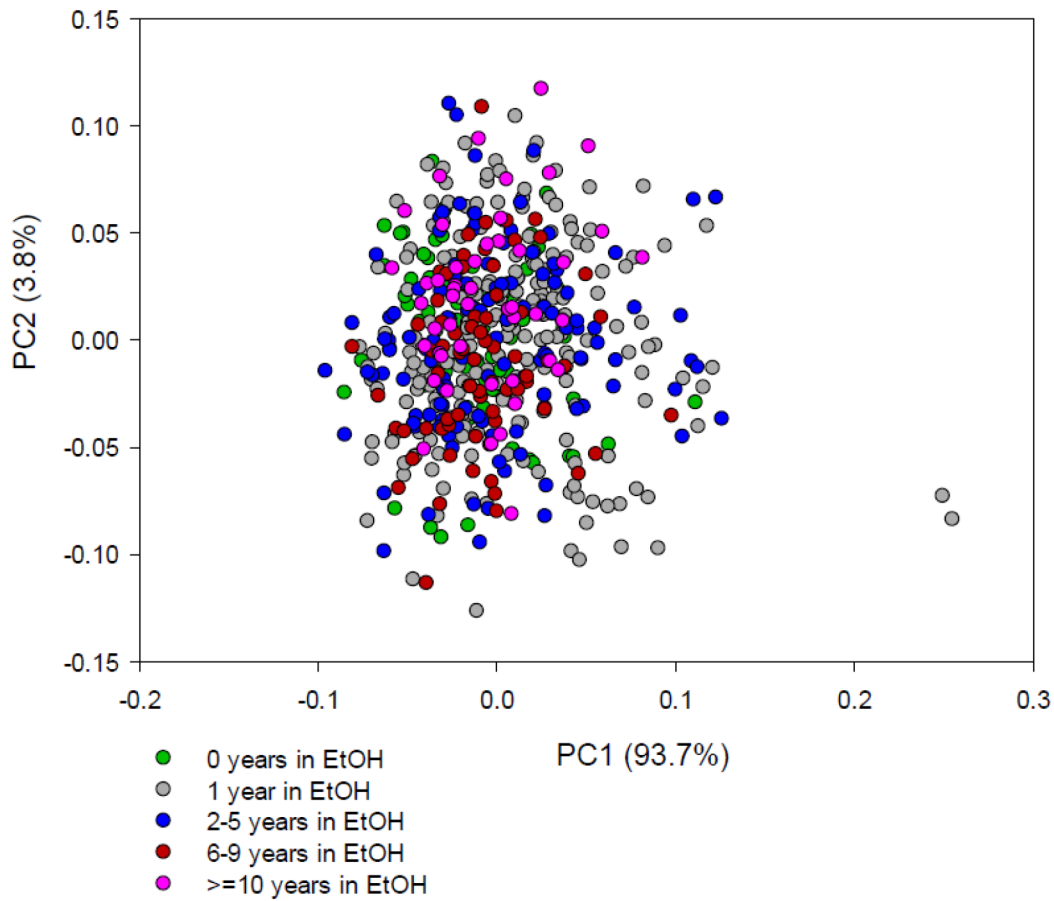

Supplement: Figure S2 — Specimens with identical ethanol storage period share the same colour. Percentage of the variation explained by each of the first two principal components (PC) given. There is no indication of clustering. [file peerj-03-991-s005.pdf]
